# Production of lentiviral vectors with enhanced efficiency to target dendritic cells by attenuating mannosidase activity of mammalian cells

**DOI:** 10.1186/1754-1611-5-1

**Published:** 2011-01-28

**Authors:** April Tai, Steven Froelich, Kye-Il Joo, Pin Wang

**Affiliations:** 1 Mork Family Department of Chemical Engineering and Materials Science, University of Southern California, Los Angeles, CA 90089, USA

## Abstract

**Background:**

Dendritic cells (DCs) are antigen-presenting immune cells that interact with T cells and have been widely studied for vaccine applications. To achieve this, DCs can be manipulated by lentiviral vectors (LVs) to express antigens to stimulate the desired antigen-specific T cell response, which gives this approach great potential to fight diseases such as cancers, HIV, and autoimmune diseases. Previously we showed that LVs enveloped with an engineered Sindbis virus glycoprotein (SVGmu) could target DCs through a specific interaction with DC-SIGN, a surface molecule predominantly expressed by DCs. We hypothesized that SVGmu interacts with DC-SIGN in a mannose-dependent manner, and that an increase in high-mannose structures on the glycoprotein surface could result in higher targeting efficiencies of LVs towards DCs. It is known that 1-deoxymannojirimycin (DMJ) can inhibit mannosidase, which is an enzyme that removes high-mannose structures during the glycosylation process. Thus, we investigated the possibility of generating LVs with enhanced capability to modify DCs by supplying DMJ during vector production.

**Results:**

Through western blot analysis and binding tests, we were able to infer that binding of SVGmu to DC-SIGN is directly related to amount of high-mannose structures on SVGmu. We also found that the titer for the LV (FUGW/SVGmu) produced with DMJ against 293T.DCSIGN, a human cell line expressing the human DC-SIGN atnibody, was over four times higher than that of vector produced without DMJ. In addition, transduction of a human DC cell line, MUTZ-3, yielded a higher transduction efficiency for the LV produced with DMJ.

**Conclusion:**

We conclude that LVs produced under conditions with inhibited mannosidase activity can effectively modify cells displaying the DC-specific marker DC-SIGN. This study offers evidence to support the utilization of DMJ in producing LVs that are enhanced carriers for the development of DC-directed vaccines.

## Background

Dendritic cells (DCs) are immune cells that are able to present antigens to T cells in a major histocompatibility complex (MHC)-restricted manner. These antigens are usually obtained by phagocytosis of pathogens encountered by the DCs [1]. The naive T cells are activated by the interaction with the antigen-presenting DCs and are then able to recognize the corresponding pathogens. To utilize this mechanism for therapeutic applications such as immunizations and vaccinations, DCs can be loaded with antigens to stimulate antigen-specific CD8+ and CD4+ T cell responses [1-4]. Another method of modifying DCs to present desired antigens is to genetically alter the cells by using liposomes or gene-gun, or by viral transduction with replication-incompetent viral vectors [5,6]. The benefits of these strategies are the increased time of antigen presentation, the ability to present both MHC I and II epitopes, and the ability to include genes for immomodulatory molecules that may enhance DC function [7]. Currently, adenoviral, gamma-retrovial, and lentiviral vectors (LVs) are studied for the viral vector delivery strategy [8-11]. LVs pose an advantage in their ability to transduce non-dividing cells, which is beneficial for *in vivo *immunization [12-16]. However, these recombinant viral vectors are known to have broad specificity and are able to transduce multiple cell types, which can inevitably result in genetic modification of undesired cells and reduce vaccine efficacy [17,18].

A surface molecule present on immature DCs, Dendritic Cell-specific ICAM3-grabbing Nonintegrin (DC-SIGN), is well-displayed and a suitable target for DC-specific transduction [18,19]. DC-SIGN is a C-type (Ca^2+^-dependent) lectin that is able to rapidly bind to and endocytose antigenic materials [20]. It is a type II transmembrane protein that is displayed as a tetramer, and consists of a short, N-terminal cytoplasmic tail that contains intracellular sorting motifs, a transmembrane region, an extracellular stalk, and a C-terminal carbohydrate-recognition domain (CRD) [21-23]. It was reported that the Sindbis virus (SV), a member of the *alphavirus *genus and the *Togaviridae *family, is able to recognize and bind to DCs through DC-SIGN [24]. However, the SV glycoprotein (SVG) also has the ability to bind to cell-surface heparin sulfate (HS), which is expressed by many cell types, and therefore LVs pseudotyped with SVG have a broad tropism [25,26]. Further studies showed that the HS binding site of SVG can be mutated [27] so that the resulting SVGmu glycoprotein can selectively recognize and bind to DC-SIGN [28]. Thus, SVGmu-pseudotyped LVs can specifically target and recognize DCs, delivering antigens that enable T cell activation for immunization and vaccine purposes [28-30].

**Figure 1:**
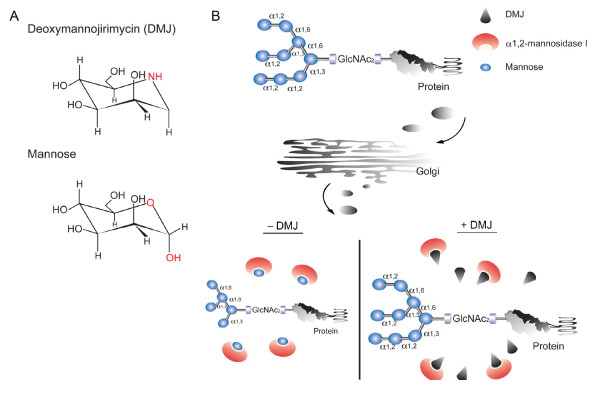
**Chemical structure of DMJ and its inhibition mechanism**. (A) Comparison of the chemical and structural composition of DMJ to that of mannose. (B) Schematic diagram of the mechanism by which DMJ inhibits class I α1,2-mannosidase in the Golgi. α1,2-mannosidase I normally trims the α1,2-linked mannose on glycoproteins; however, DMJ inhibits α1,2-mannosidase I activity and thus restricts the oligosaccharide processing to high-mannose forms.

The study of DC-SIGN binding to other proteins has shown that binding occurs in a carbohydrate-dependent manner [20,31]; in fact, Sindbis viruses produced in mosquito cells, which limit glycoprotein processing and carbohydrate trimming, yielded higher transduction efficiencies for DC-SIGN-bearing cells compared to viruses produced in mammalian cells [24]. The high-mannose structures on gp120 have also been studied and have been determined to be critical for recognition to DC-SIGN [32,33]. Mannosidase is a calcium-dependent enzyme that removes mannose from N-linked glycoproteins in the ER and Golgi [34]. 1-deoxymannojirimycin (DMJ, Figure 1A) is a chemical that can inihibit α-mannosidase I in the Golgi by binding to the top of its C-terminal α-hairpin, which is located at the bottom of the active site cavity [34-36]. This effectively halts the processing of the oligosaccharide at Man_9_GlcNAc_2 _(Figure 1B) [37]. It has been reported that DC-SIGN binds to Man_9_GlcNAc_2 _130-fold more tightly than it does to mannose [31]. Thus, we hypothesized that SVGmu also binds to DC-SIGN through high-mannose structures, and that the addition of DMJ inhibits the activities of α1,2-mannosidase I, which would allow for a greater amount of high-mannose structures on the surface of SVGmu-pseudotyped LVs. In this study, we test this hypothesis and show that SVGmu-bearing LVs produced under DMJ treatment can modify cells expressing the DC-specific marker DC-SIGN more efficiently.

## Results and Discussion

### Transient transfection of 293T cells to produce SVGmu-pseudotyped LVs

**Figure 2:**
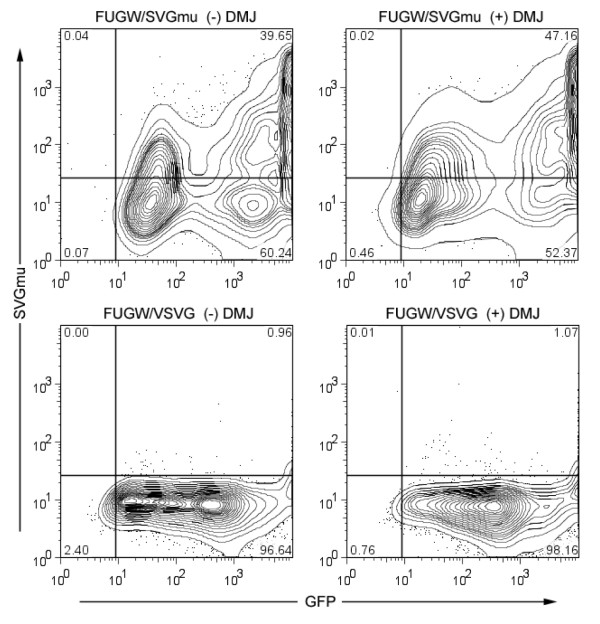
**DMJ does not reduce SVGmu production or display on vector-producing cells**. 293T cells were transiently transfected by the lentiviral backbone vector encoding the GFP gene (FUGW), packaging constructs (REV and RRE), and a plasmid encoding either SVGmu or VSVG. SVGmu-staining and flow cytometry analysis of the cells two days post-transfection revealed that cells cultured with or without DMJ exhibited similar levels of SVGmu and GFP, indicating that the presence of DMJ did not restrict either glycoprotein or LV production. Control cells transfected by VSVG were similarly unaffected by DMJ and SVGmu-negative, as expected.

To assess the viability of producing LVs in media containing DMJ, 293T cells were transiently transfected with a lentiviral backbone plasmid (FUGW) encoding a green fluorescent protein (GFP) reporter gene driven by the human ubiqutin-C promoter [38], packaging plasmids, and a plasmid encoding either SVGmu or vesicular stomatitis virus glycoprotein (VSVG). VSVG has widely been used to pseudotype LVs and the resulting vectors have a very broad tropism [26]. Thus, we included VSVG in our transfection to produce a control vector that is not DC-SIGN-targeting. Analysis of the transfected cells two days later by flow cytometry showed comparable results between the samples with and without DMJ added, with slightly higher values for SVGmu-staining in the cells transfected with SVGmu and cultured with DMJ (Figure 2). Cells transfected with SVGmu stained positively for SVGmu and expressed GFP, while the control cells transfected with VSVG were only GFP-positive. These results indicate that the addition of DMJ to the cell culture media does not adversely affect the transfection of 293T cells.

### Verification of SVGmu and high-mannose oligosaccharides on the vector surface

**Figure 3:**
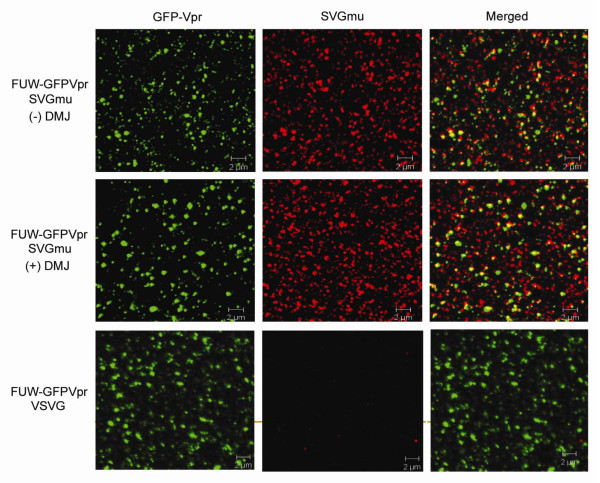
**SVGmu is incorporated onto the vector surface for LVs produced with or without DMJ**. Fresh viruses were produced with the addition of an additional plasmid, GFP-Vpr to fluorescently label the vector core. Vectors produced with or without DMJ were then stained for SVGmu and analyzed by confocal microscopy. Visualization of the vector particles showed that SVGmu was efficiently incorporated in both types of vectors. VSVG-pseudotyped vectors were included as a control.

To verify the presence of SVGmu on the vector particle surface produced in media containing DMJ, we employed a labeling scheme to generate vectors that encapsulated GFP-Vpr (GFP fused with the HIV accessory protein Vpr [39]). A transient co-transfection protocol similar to what was described above, was utilized to generate the GFP-labeled particles, except that the lentiviral backbone plasmid FUW, which lacks the GFP reporter gene, replaced FUGW, and an additional plasmid encoding GFP-Vpr was included in the co-transfection procedure [39]. The resulting vectors (FUW-GFP-Vpr/SVGmu +/- DMJ or FUW-GFP-Vpr/VSVG) were loaded onto cover slips, stained for SVGmu, and analyzed by confocal imaging. The SVGmu envelope glycoprotein was confirmed to be displayed on the FUW-GFPVpr/SVGmu vectors produced both with and without DMJ by the colocalization of the GFP and SVGmu signals (Figure 3). As a control, FUW-GFPVpr/VSVG vector particles were also analyzed. As expected, while these particles were GFP-positive, they did not stain for SVGmu.

**Figure 4:**
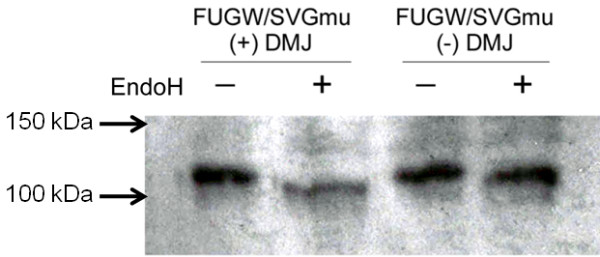
**LVs produced in DMJ contain more high-mannose structures**. Vectors were produced with or without DMJ, concentrated, and digested by EndoH, an enzyme that cleaves high-mannose structures. A western blot analysis of the undigested and digested vectors showed that the vectors produced in DMJ had a lower molecular weight after EndoH digestion compared to that of the vectors produced without DMJ. These results infer that the vector produced with DMJ had more high-mannose structures than the vector produced without DMJ.

Next, the SVGmu-enveloped vector (FUGW/SVGmu) produced either with or without DMJ was concentrated by ultracentrifugation and then digested by EndoH, an enzyme that selectively breaks apart high-mannose structures by cleaving the chitobiose core from N-linked glycoproteins [20]. A western blot analysis of the EndoH-treated vector particles confirmed the presence of high-mannose structures for the LVs produced with DMJ by yielding a lower molecular weight species (Figure 4). In contrast, the vector produced without DMJ did not give as low of a molecular weight species when digested by EndoH, which is evidence of less high-mannose structures present on the vector surface. We believe that this supports our hypothesis that the untreated vector contains mostly complex sugars on the envelope glycoprotein, along with some hybrid sugars (both mannose and complex sugars) and/or high-mannose sugars.

### Evaluation of cell-vector binding to DC-SIGN

**Figure 5:**
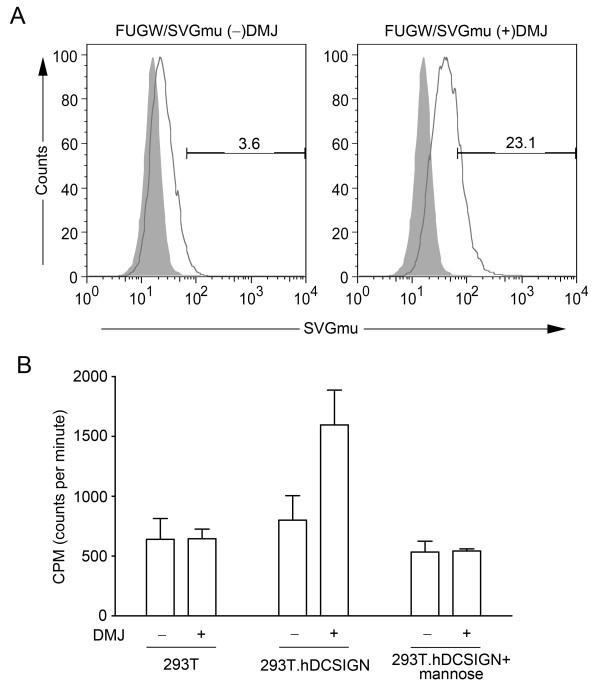
**LVs produced in DMJ bind more readily to human DCSIGN than vectors produced without DMJ**. (A) Vectors produced with or without DMJ were incubated with fixed 293T.DCSIGN cells, a cell line that stably displays human DCSIGN. Flow cytometric analysis of SVGmu-stained cells revealed that the vectors produced in DMJ bound to the DCSIGN-expressing cells over 6 times more readily than the vectors produced without DMJ. (B) Vectors were labeled with [35S]-Trans and incubated with either 293T or 293T.DCSIGN cells. A mannose inhibition assay was also included to determine the dependency of vector-receptor binding on the mannose-rich structures on the vector. Radioactivity analysis revealed that the vector produced with DMJ bound much more readily to the 293T.DCSIGN cells than to the 293T cells, while the addition of mannose greatly reduced cell-vector binding. Vectors produced without DMJ did not exhibit as much of a difference between binding to the 293T.DCSIGN cells and the 293T cells, while the presence of mannose reduced vector levels in both of the cell lines.

Two experiments were performed to investigate whether SVGmu does in fact recognize and bind to human DC-SIGN and whether binding of SVGmu-bearing LVs (FUGW/SVGmu) to 293T.DCSIGN, a stable 293T cell line expressing human DCSIGN, is enhanced by the addition of DMJ to vector-producing cells. In the first experiment, 293T.DCSIGN cells were fixed with 4% paraformaldehyde followed by incubation with fresh supernatant containing FUGW/SVGmu. The cell-vector complexes were then stained for SVGmu and analyzed by flow cytometry. As expected, the vector produced with DMJ bound more readily to the cells, as shown by the higher percentage of SVGmu staining (Figure 5A).

Next, a radioactive-labeling assay was used to test the cell-vector binding responses for LVs produced with and without DMJ, with an additional comparison to control 293T cells, which lacked DC-SIGN expression. 293T and 293T.DCSIGN cells were seeded onto 96-well plates overnight. The cells were then washed by PBS and incubated with concentrated, ^35^S-labeled LVs for one hour. A second wash with PBS removed unbound vectors and the cells were lysed before they were pipetted into scintillation vials for analysis. A mannose inhibition assay was also employed to test the dependency of vector binding on the mannose-rich structures of the envelope glycoprotein. Mannose was incubated with the cells prior to the addition of LVs to block the binding sites that are mannose-linked. A much higher amount of radioactivity was detected in FUGW/SVGmu(DMJ+) incubated with 293T.DCSIGN as compared to FUGW/SVGmu(DMJ-) (Figure 5B). In contrast, DMJ did not greatly alter the binding ability of FUGW/SVGmu to 293T cells; vectors produced with and without DMJ resulted in a low incidence of binding. Although the addition of mannose lowered the amount of cell-vector binding measured with both types of LVs (DMJ+ and DMJ-) and both types of cells (293T.DCSIGN and 293T), it had the most significant effect on the binding of FUGW/SVGmu(DMJ+) to the 293T.DCSIGN cells. This data suggests that the enhanced ability of the LV to bind to DC-SIGN we observed was the result of its greater availability of high-mannose structures, and that this interaction can be blocked by competitive inhibition with mannose.

### Transduction of cells with LVs produced with DMJ

**Figure 6:**
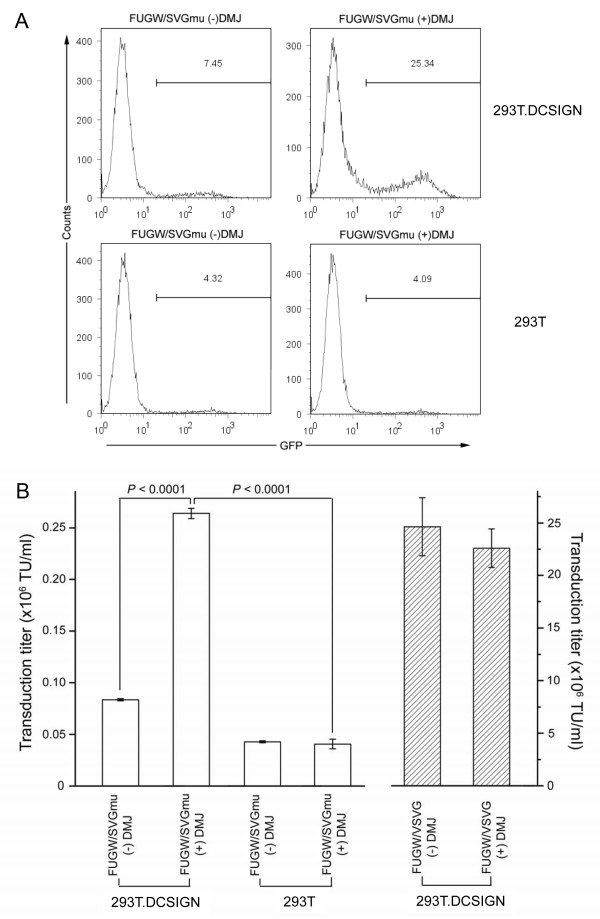
**LVs produced with DMJ transduced DCSIGN-expressing cells much more efficiently than LVs produced without DMJ**. (A) LVs were produced either with or without DMJ and spin-infected with 293T and 293T.DCSIGN cells. Although FUGW/SVGmu(DMJ-) preferentially transduced 293T.DCSIGN cells, FUGW/SVGmu(DMJ+) was over three-fold more efficient in transducing 293T.DCSIGN cells while maintaining similar levels of background transduction to 293T cells. (B) LVs produced with DMJ yielded higher vector titers for DCSIGN-expressing cells compared with LVs produced without DMJ. Vector titers for both FUGW/SVGmu +/- DMJ were analyzed for 293T.DCSIGN and 293T cells. On 293T.DCSIGN cells, FUGW/SVGmu(DMJ+) yielded titers over three-folds higher than those of FUGW/SVGmu(DMJ-), while titers for 293T cells for both vectors were similarly low. FUGW/VSVG +/- DMJ was included as a control.

LVs produced with DMJ were used to transduce 293T and 293T.DCSIGN cells to test the effect of the improved binding on cell transduction. Fresh vector supernatants were added to cells of each type, followed by spin-infection. The cells were then analyzed for GFP expression by flow cytometry after three days of incubation. A p24 ELISA assay was also performed to ensure that the concentrations of the different viruses were comparable. FUGW/SVGmu(DMJ+) transduced 293T.DCSIGN cells with the greatest efficiency (25.34%) - more than three-folds higher than the FUGW/SVGmu(DMJ-) vector (7.45%) (Figure 6A). Both types of LVs had a similar amount of transduction for the control 293T cells. These results validate our expectation that more highly infective targeting vectors can be produced with the addition of DMJ in the cell culture media during vector production.

Vector titers were then calculated for FUGW/SVGmu +/- DMJ against 293T and 293T.DCSIGN cells. FUGW/VSVG +/- DMJ were also included as controls. Triplicate experiments were performed, where cells were spin-infected with various fresh vector supernatants that had been serially diluted. Three days later, the cells were washed and analyzed for GFP expression by flow cytometry. As expected, FUGW/SVGmu(DMJ+) yielded a higher transduction titer with 293T.DCSIGN cells, with an average value of 0.26 × 10^6 ^TU/ml, as compared to FUGW/SVGmu(DMJ-), which had an average transduction titer of 0.08 × 10^6 ^TU/ml (Figure 6B). FUGW/SVGmu +/- DMJ both transduced 293T cells similarly with titers of ~0.04 × 10^6 ^TU/ml. As an additional control, 293T.DCSIGN cells were also tranduced by FUGW/VSVG +/- DMJ and titers of 22.60 × 10^6 ^TU/ml and 24.60 × 10^6 ^TU/ml, respectively, were obtained. Thus, our titer measurement confirms that FUGW/SVGmu(DMJ+) transduced 293T.DCSIGN cells three times more efficiently than FUGW/SVGmu(DMJ-) did, and that both FUGW/SVGmu +/- DMJ transduced 293T cells at an equally low rate. The addition of DMJ to the production of the FUGW/VSVG vector did not significantly alter the vector titer against 293T.DCSIGN.

One group demonstrated that LVs pseudotyped with a similarly modified Sindbis virus envelope glycoprotein produced without DMJ treatment did not bind to DC-SIGN and target DC-SIGN-positive cells [40]. The mutations used in their envelope protein were identical except for the addition of a ZZ domain versus the HA tag employed in our system. However, previous studies in our laboratory have shown that SVGmu binds with DC-SIGN and targets transduction to DC-SIGN-positive cells [28]. Also, compared with our data for wild-type Sindbis envelope-bearing LVs, their pseudotyped vectors are less infectious (Virus-receptor mediated transduction of dendritic cells by lentiviruses enveloped with glycoproteins derived from Semliki Forest virus and Ross River virus, submitted). Perhaps these differences in infectious vector production are indicative of an abundance of non-infectious particles that conceal interactions between the glycoprotein and DC-SIGN. Other differences, such as our use of a clonally-expanded DC-SIGN-expressing cell line and other experimental settings, may further contribute to the reported inability to observe increased transduction efficiencies for DC-SIGN bearing cell lines with SVGmu envelope-bearing LVs produced without DMJ.

### Transduction of a DC cell line with vectors produced with DMJ

**Figure 7:**
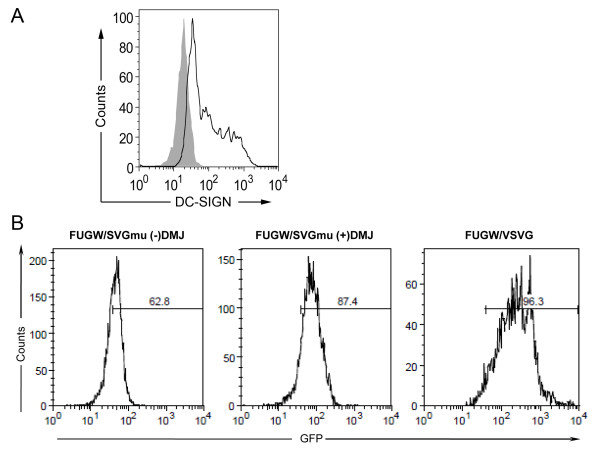
**LVs produced with DMJ can transduce a DC cell line more efficiently than LVs produced without DMJ**. A human DC cell line (MUTZ-3) was employed to more accurately model human DCs, as a target for LVs produced with and without DMJ. (A) MUTZ-3 cells were differentiated for 7 days and stained for DC-SIGN expression. (B) The MUTZ-3 cells were transduced by LVs produced with or without DMJ. FUGW/SVGmu(DMJ+) transduced the MUTZ-3 cells about 25% more efficiently than FUGW/SVGmu(DMJ-). FUGW/VSVG was included as a positive control.

To test the transduction efficiency of vectors produced with and without DMJ on a closer model of dendritic cells, we utilized a DC cell line (MUTZ-3 cells) that had been previously shown to closely mirror the behavior of human dendritic cells and are capable of being differentiated to express human DC-SIGN [41,42]. MUTZ-3 cells were cultured and differentiated for 7 days to express DC-SIGN (Figure 7A) before they were transduced by concentrated FUGW/SVGmu vectors produced with or without DMJ. Consistent with the results from 293T.DCSIGN transduction, the targeting vector produced with DMJ transduced the MUTZ-3 cells more efficiently (87.4%) than the vector produced without DMJ (62.8%) (Figure 7B). Thus, the targeting vector produced with DMJ, which contained more high-mannose structures, was able to transduce both the 293T.DCSIGN cell line and a human DC cell line more efficiently than the vector produced without DMJ.

## Conclusions

DC-based vaccines have great potential as a new means to fight challenging diseases, such as cancers, HIV, and autoimmune diseases. Optimization of the efficiency of the viral vectors used to modify DCs could greatly strengthen this approach and make it an even more powerful tool for developing novel treatment modalities against various diseases. Our study aimed to examine the effects of DMJ, which indirectly increases the amount of high-mannose structures present on glycoproteins through the inhibition of class I α1,2-mannosidase activity in vector-producing cells, on the efficiency of SVGmu-bearing LV transduction of DC-SIGN-expressing cells.

We were able to conclude that the FUGW/SVGmu vector made in the presence of DMJ did in fact have increased high-mannose structures over those made without DMJ present. Our results also suggested that binding of SVGmu to DC-SIGN is directly related to amount of high-mannose structures on SVGmu, and that these interactions can be blocked by competitive inhibition. Furthermore, we found that production of the targeting vector in DMJ resulted in a three-fold increase in transduction efficiency in target cells compared to vectored produced without DMJ. Lastly, the targeting vector produced in the presence of DMJ was able to transduce MUTZ-3 cells, a cell line that has been shown to closely mimic the behavior of human peripheral blood mononuclear cells (PBMCs) and can be differentiated to cells displaying DC-SIGN, more efficiently than the vector produced without DMJ, which strengthens our belief that an increase in high-mannose structures on the targeting virus surface would result in higher transduction efficiency of human DCs.

Further investigation into the vector entry mechanism of SVGmu-pseudotyped LVs is currently being conducted using confocal microscopy with the aid of drug treatments. Greater understanding of the pathway of viral uptake and core release into the cytosol will facilitate the design of more effective and efficient engineered gene delivery vehicles. Additionally, siRNA can be applied to silence mannosidase in vector-producing cells to eliminate the need for DMJ in large-scale productions of optimized high-mannose-containing engineered LVs.

## Methods

### Production of lentiviral vectors

293T cells were transiently transfected with a standard calcium phosphate precipitation protocol. 293T cells were seeded onto 6-cm tissue culture dishes and transfected with 5 μg of the lentiviral transfer vector plasmid (FUGW or FUW), 2.5 μg of the envelope plasmid (SVGmu or VSVG), and packaging plasmids (pMDLg/pRRE and pRSV-Rev). D10 media (Dulbecco's modified Eagle's medium (Mediatech Inc., Manassas, VA) with 10% fetal bovine serum (Sigma, St. Louis, MO), 2 mM L-glutamine (Hyclone, Logan, UT), 100 U/ml penicillin, and 100 μg/ml streptomycin) with and without DMJ was replaced 4 h later. After 48 h post-transfection, the vector supernatants were harvested and filtered through a 0.45-μm filter (Corning, Acton, MA). To concentrate the vector, the supernatants were ultracentrifugated (Optima L-80 K preparative ultracentrifuge, Beckman Coulter, Brea, CA) after filtration at 50,000 × g for 90 min. The viral pellets were resuspended in 100 μl of cold PBS.

### Confocal imaging of lentiviral vectors

LVs were produced with the addition of 2.5 μg of a plasmid encoding GFP-Vpr. Vector supernatant was placed on polylysine-coated coverslips in 6-well culture dishes and centrifuged at 3,700 × g at 4°C for 2 h with a Sorvall Legend RT centrifuge (DJB Labcare, Buckinghamshire, England). The coverslips were then washed twice with cold PBS and immunostained with anti-HA-biotin antibody (Miltenyi Biotec, Bergisch Gladbach, Germany) and Cy5-streptavidin (Invitrogen, Carlsbad, CA). The samples were fluorescently imaged by a Zeiss LSM 510 laser scanning confocal microscope with filter sets for fluorescein and Cy5 and a plan-apochromat oil immersion objective (63×/1.4).

### Vector digestion by EndoH and western blot

7 μl of concentrated virus was combined with 2 μl of lysis buffer and incubated at 37°C for 30 min. Next, the glycoprotein was denatured by adding 1 μl of 10× Glycoprotein Denaturing Buffer to the mixture and heating the reaction to 100°C for 10 min. The protein was then digested by EndoH by adding 2 μl of 10 × G5 Reaction Buffer, 5 μl of EndoH (NEB, Ipswich, MA), and 3 μl of H_2_O. This reaction was incubated at 37°C for 1 h. Protein gels were run and then western blots were performed to transfer the proteins onto membranes. The membranes were then blocked by 5% milk with TBST at 4°C for 1 h. The membranes were washed with TBST and then stained for anti-HA-biotin antibody for 1 h. They were then washed again, stained for streptavidin-HRP (R&D Systems, Minneapolis, MN) for 1 hr, and washed. To develop the western blot, TMB solution was spread onto the surface of the membrane and left for 10 min at room temperature. The membrane was then imaged.

### Radioactive labeling and mannose inhibition of lentiviral vectors

293T cells were transfected by vector plasmids as described earlier, without the addition of DMJ. 20 h post-transfection, the media was replaced with methionine-free D10 with or without DMJ. After 4 h, 50 μl of [35S]-Trans (MP Biochemicals, Solon, OH) was added to 15-cm tissue culture dishes and incubated at 37°C. 48 h post-tranfection, viral supernatants were harvested, filtered, and concentrated. For the mannose inhibition assay, 10 mM of D-mannose was added to the 293T.hDCSIGN cells prior to the cell-virus binding tests.

### Vector transduction of cells

293T or 293T.DCSIGN cells were seeded into a 24-well culture dish at 0.2 × 10^6 ^cells per well and spin-infected with 1 ml of viral supernatant per well at 2,500 rpm and 25°C for 90 min using a Sorvell Legend centrifuge. The cell supernatants were then replaced with fresh D10 media and incubated at 37°C for 3 days with 5% CO_2_. FACS analysis was used to determine the percentage of GFP+ cells present. To determine transduction titers, the dilution ranges that showed a linear response were used.

### Transduction of a human DC cell line

MUTZ-3 cells (Deutsche Sammlung von Mikroorganismen und Zellkulturen, Braunschweig, Germany) were cultured in 24-well tissue culture plates in αMEM media (BioWhittaker, Walkersville, MD) with 20% FBS (Sigma-Aldrich, St. Louis, MO) and 40 ng/mL GM-CSF (PeproTech, Rocky Hill, NJ). To differentiate the cells to express human DC-SIGN, MUTZ-3 cells were cultured in the presence of IL-4 and GM-CSF (100 ng/mL of each; PeproTech) for 7 days. FACS analysis of cells stained with anti-human DCSIGN-PE (BioLegend, San Diego, CA) confirmed the presence of the human DC-SIGN marker. The cells (1 × 10^5^) were spin-infected with concentrated virus and the medium was replaced with fresh medium containing IL-4 and GM-CSF. The cells were analyzed by flow cytometry for GFP expression 3 days post-tranduction.

## Abbreviations

DC): dendritic cell; (DMJ): 1-deoxymannojirimycin; (FUGW): lentiviral vector carrying a GFP reporter gene under the control of the human ubiquitin C promoter; (GFP): green fluorescent protein; (GFPVpr): GFP fused to the N-terminus of Vpr; (HS): heparin sulfate; (LV): lentiviral vector; (MHC): major histocompatibility complex; (SVG): Sindbis virus glycoprotein; (SVGmu): engineered SVG; (Vpr): the HIV accessory protein viral protein R; (VSVG): vesicular stomatitis viral glycoprotein.

## Competing interests

The authors declare that they have no competing interests.

## Authors' contributions

AT and PW designed experiments. AT, SF, and KJ carried out all the experiments. AT and PW wrote the manuscript. All authors read and approved the final manuscript.
